# Superinfection exclusion is absent during acute Junin virus infection of Vero and A549 cells

**DOI:** 10.1038/srep15990

**Published:** 2015-11-09

**Authors:** Raphaël Gaudin, Tomas Kirchhausen

**Affiliations:** 1 Department of Cell Biology, Harvard Medical School and Program in Cellular and Molecular Medicine, Boston Children’s Hospital, 200 Longwood Ave, Boston, MA 02115, USA; 2 Department of Pediatrics, Harvard Medical School, 200 Longwood Ave, Boston, MA 02115, USA

## Abstract

Many viruses have evolved strategies of so-called “superinfection exclusion” to prevent re-infection of a cell that the same virus has already infected. Although Old World arenavirus infection results in down-regulation of its viral receptor and thus superinfection exclusion, whether New World arenaviruses have evolved such a mechanism remains unclear. Here we show that acute infection by the New World Junin virus (JUNV) failed to down-regulate the transferrin receptor and did not induce superinfection exclusion. We observed that Vero cells infected by a first round of JUNV (*Candid1* strain) preserve an ability to internalize new incoming JUNV particles that is comparable to that of non-infected cells. Moreover, we developed a dual infection assay with the wild-type *Candid1* JUNV and a recombinant JUNV-GFP virus to discriminate between first and second infections at the transcriptional and translational levels. We found that Vero and A549 cells already infected by JUNV were fully competent to transcribe viral RNA from a second round of infection. Furthermore, flow cytometry analysis of viral protein expression indicated that viral translation was normal, regardless of whether cells were previously infected or not. We conclude that in acutely infected cells, Junin virus lacks a superinfection exclusion mechanism.

Arenaviruses are enveloped viruses with two segments of an ambisense single-stranded RNA genome. Some of these viruses cause hemorrhagic fever with poor prognoses in humans, including the New World (NW) arenavirus (clade B) Junin virus (JUNV), which is responsible for Argentine hemorrhagic fever^1^. An attenuated strain, *Candid1*, shows strong protection efficacy in humans^2^ and is used to study the life cycle of NW arenaviruses.

Many viruses have evolved strategies to prevent superinfection of acutely^3^,^4^,^5^,^6^,^7^ or chronically^8^,^9^,^10^ infected cells. The likely role of this mechanism, also known as superinfection exclusion, may be to avoid competition for resources. Vero cells acutely infected with *Candid1* are permissive for a second round of infection with the alphavirus Venezuelan equine encephalitis virus (VEEV), probably because they are interferon-deficient^7^; in contrast, A459 cells similarly infected with *Candid1* are resistant to a second round of infection with VEEV presumably due to induction of a potent type-I interferon response^7^. Old World (OW) arenavirus infection leads to the down-modulation of its viral receptor α-dystroglycan^11^, although superinfection exclusion has not been directly assessed in this study. In the case of NW arenaviruses, Ellenberg *et al.* reported that Vero cells chronically infected with JUNV are not permissive to a second round of homologous JUNV infection^12^. The authors concluded that superinfection exclusion was in part the result of a defect in viral RNA replication of the second JUNV genome. In contrast, chronically JUNV-infected BHK-21 cells are permissive to the early stages of a superinfection, but deficient for viral assembly and release^13^. The superinfection exclusion described in those two studies was characterized in a model of chronic infection, but whether it occurs during the acute phase of JUNV infection remains to be determined.

Here, we show that superinfection exclusion does not occur during acute sequential rounds of infection of either Vero or A549 cells with the *Candid1* strain of JUNV. Cells acutely infected by a first round of JUNV infection are still fully permissive for virus internalization, viral RNA synthesis, and translation of viral proteins associated with a second round of JUNV infection harbouring the same *Candid1* surface glycoprotein complex (GPC). To the best of our knowledge, these results indicate that JUNV is one of the only viruses that does not exhibit superinfection exclusion by its own kind.

## Results and Discussion

**Figure 1:**
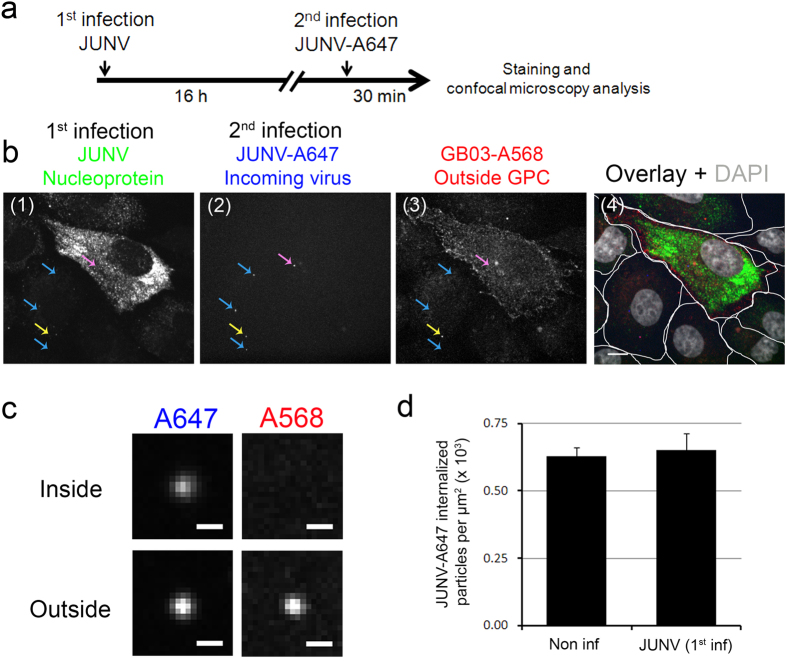
Junin virus particle internalization during superinfection. (**a**) Experimental design. (**b**) The representative images correspond to the fluorescence channels obtained from a maximum projection of a Z-stack of consecutive planes imaged 500 nm apart. Subpanel 1 highlights the difference in NP staining between a cell infected during the first round of infection and the remaining non-infected cells. Subpanel 2 shows examples of fluorescently tagged viral particles associated with cells during the second round of infection. Subpanel 3 shows the relatively diffuse staining of GPC of a cell infected during the first round of infection and the punctae corresponding to viral particles adsorbed to the cell surface during the first and second rounds of infection. The yellow arrow corresponds to a non-internalized virus adsorbed to the surface of a non-infected cell; the blue arrows highlight viruses internalized by a cell that was not infected during the first round of infection; and the pink arrow indicates a virus internalized by a cell infected during the first round of infection. Subpanel 4 depicts the overlay of all channels and highlights the outline of all of the cells in the field. The images correspond to a Z-stack maximum projection of consecutive planes imaged 500 nm apart. Scale bar: 10 μm. (**c**) Example of images comparing the fluorescence signal for an internalized JUNV particle (“inside” upper panel, signal for JUNV-A647 but not for GB-A568) and a JUNV particle that remained bound to the cell surface (“outside” lower panel, signals for JUNV-A647 and GB-A568) in cells infected during the first round. Scale bar: 1 μm. (**d**) Quantitative comparison of the number of JUNV particles internalized during the second round of infection between cells that were or were not infected during the first round of infection. The data are expressed as the count of internalized JUNV-A647 normalized to the projected area of the cells. Data are average ± SD from two independent experiments in which more than 200 particles were counted.

We first used a fluorescence microscopy visualization assay to determine whether the JUNV-infected cells allow internalization of new, incoming viral particles (Fig. 1). Entry of fluorescently tagged Junin virus into single cells was assessed using spinning disc confocal fluorescence microscopy according to the experimental design summarized in Fig. 1a. Vero cells were infected at a multiplicity of infection (MOI) of 0.1 and superinfected 16 h later with JUNV particles complexed to an Alexa Fluor 647–labelled non-neutralizing antibody^14^,^15^ to allow visualization of the cell-associated virus particles related to the second round of infection. To discriminate virus particles bound to the cell surface (Fig. 1c, outside) from those that were internalized (Fig. 1c, inside), cells were fixed and incubated without permeabilization with an Alexa Fluor 568–tagged monoclonal antibody specific for the virus glycoprotein complex (GPC) (GB03-A568, outside GPC). After an extensive washing to remove unbound antibodies, cells were fixed and permeabilized, and the nucleoprotein (NP) was detected using an A488-tagged monoclonal antibody. Cells infected during the first round of infection showed extensive and diffuse cytosolic fluorescence NP signal whereas cells infected only during superinfection showed punctae corresponding to bound or internalized particles (Fig. 1b). The relative number of particles associated with superinfected cells was obtained from maximum intensity Z-projections of consecutive optical sections spanning the entire cell volume imaged 500 nm apart and normalized by the area of the cell (Fig. 1d). These results demonstrate that pre-infection of Vero cells did not affect the entry of JUNV particles during superinfection.

**Figure 2:**
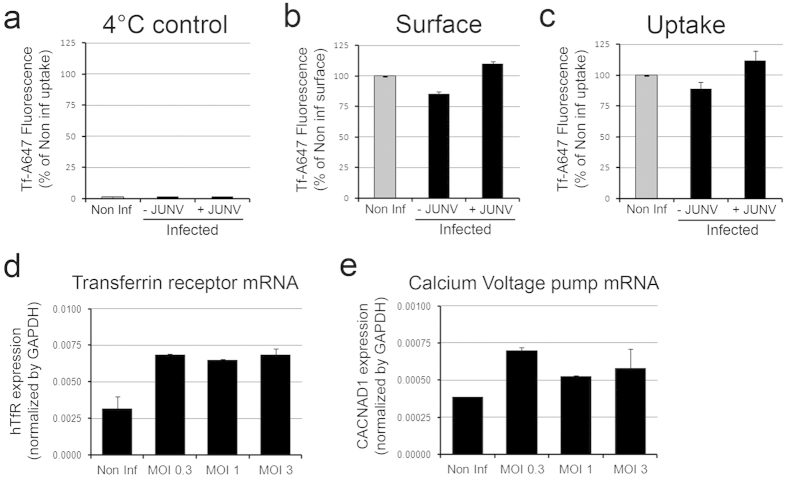
Amounts of transferrin receptor and calcium voltage pump and endocytosis of transferrin in Vero cells acutely infected with JUNV. Vero cells were grown for 16 h in the absence (non Inf) or presence (Infected) of JUNV and then analysed for (**a–c**) the surface distribution of transferrin receptor and endocytosis of transferrin and (**d,e**) for the cellular expression of mRNAs encoding transferrin receptor and the calcium voltage pump. (**a–c**) The surface expression of transferrin receptor (determined by bound Alexa Fluor 647-tagged transferrin (Tf-A647)) and the receptor-mediated endocytosis of Tf-A647 were determined using a flow cytometry assay. Vero cells infected or not with JUNV using and MOI of 0.5 were incubated with Tf-A647 for 10 min at 4 °C (**a,b**) or 37 °C (**c**) and washed with a low pH buffer (**a,c**) or PBS (**b**). After fixation and permeabilization, the cells were stained with the mouse monoclonal antibody SA02-A488 specific for the JUNV nucleoprotein NP to distinguish between non-infected (– JUNV) and infected (+JUNV) cells. Data correspond to the average ± SD of two independent experiments from at least 10,000 cells per condition. Minimal fluorescence signal shown in (**a**) indicates full removal of surface bound transferrin by the acid wash step (negative control). The fluorescence signal in (**b**) is proportional to the amount of transferrin receptor at the cell surface whereas the signal in (**c**) reflects internalized transferrin. (**d,e**) Expression levels of mRNA encoding transferrin receptor and the calcium voltage pump of Vero cells infected for 16 h with JUNV at the indicated MOIs were determined by reverse transcription RT-qPCR using primers specific for the human TfR1 and CACNA2D1. The mRNA expression levels were normalized by GAPDH expression, and histograms represent mean ±SD of triplicates.

The human transferrin receptor (TfR) is considered the main receptor for Junin virus^16^. Here, we investigated whether acute JUNV infection of Vero cells could down-modulate the amount of available TfR. We found that the amount of TfR expressed at the cell surface was not affected by 16 h of infection (Fig. 2b). Consistent with this, the efficiency of receptor-mediated endocytosis of Tf (Fig. 2c) remained stable. Transferrin receptor mRNA levels as well as those of the newly described JUNV entry factor calcium-voltage pump^17^ were significantly increased, independently of the MOI used (Fig. 2d,e). Consistent with our results, CD34^+^ hematopoietic progenitor cells infected with JUNV for 120 h have also been shown to express higher TfR levels^18^.

**Figure 3:**
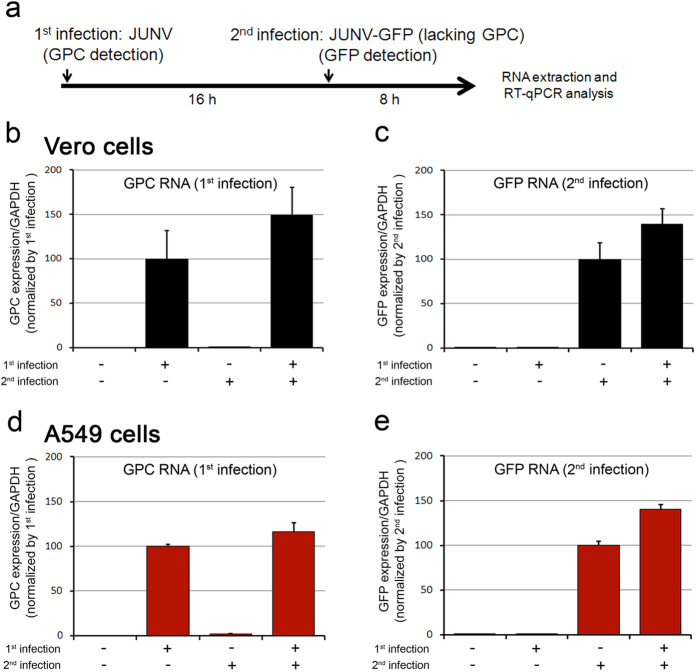
Transcription of the viral genome from a second round of infection is not affected by a first acute JUNV infection. (**a**) Experimental design. Histograms represent GPC detection corresponding to the first infection (**b**,**d**) and GFP detection corresponding to the second infection (**c**,**e**) from Vero cells (**b,c**) or A549 cells (**d,e**). Data are mean ± SD from two independent experiments performed in triplicate.

The Z protein of the Tacaribe virus, another NW arenavirus closely related to JUNV, inhibits viral RNA synthesis by direct interaction with the L viral polymerase^19^. To determine whether superinfecting JUNV might also inhibit its own replication, we used as a marker of infection a recombinant JUNV that expresses enhanced green fluorescent protein (GFP)^20^. JUNV-GFP contains the *Candid1* short segment, in which the GPC gene was replaced with GFP, so that its growth in BSR-T7 cells is complemented in *trans* by expression of *Candid1* GPC. The cells producing JUNV-GFP were also infected with a recombinant vaccinia virus encoding T7 polymerase (vTF7-3) because the recombinant JUNV genome is expressed under the control of a T7 promoter^21^. Vero cells were first infected with JUNV at a MOI of 5 for 16 h and then superinfected with JUNV-GFP for an additional 8 h. Cells were then lysed and RNA extracted for reverse-transcription real-time quantitative PCR (RT-qPCR; see experimental design in Fig. 3a). Because the recombinant JUNV-GFP virus does not contain the sequence coding for GPC, GPC RNA detection by RT-qPCR was used to monitor the first round of infection with JUNV (Fig. 3b). RT-qPCR specific for the GFP sequence was used to evaluate the efficiency of replication in the second round of infection with JUNV-GFP (Fig. 3c). The results showed that a similar amount of GFP RNA from JUNV-GFP was generated in the second round regardless of a first round of infection with JUNV.

Because Vero cells are deficient for interferon α and β production, we also tested superinfection in the interferon-competent A549 cell line (Fig. 3d,e) and confirmed that GFP RNA expression from the second infection was identical whether or not the cells were previously infected with JUNV, further suggesting an absence of superinfection exclusion as measured by the amount of replication during acute JUNV infection.

**Figure 4:**
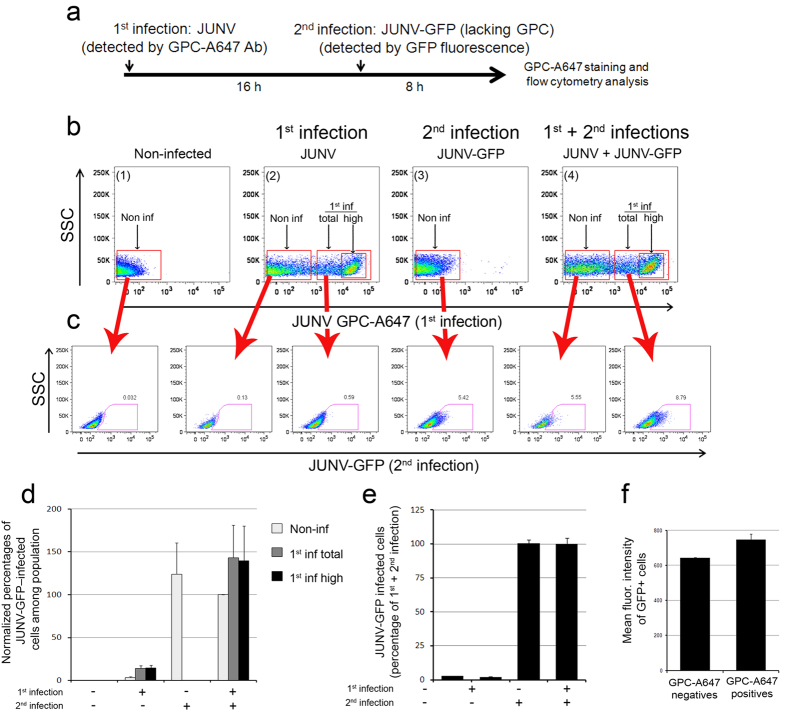
Translation of viral proteins from a second round of infection is not affected by a first acute JUNV infection. (**a**) Experimental design. (**b**) Representative examples of fluorescence scatter plots and the regions selected for analysis. The negative control in subpanel 1 corresponds to cells that had not been infected. Subpanel 2 corresponds to cells analysed 8 h after the 16-h infection period with JUNV only. Subpanel 3 corresponds to cells incubated only with JUNV-GFP during the last 8-h period. Subpanel 4 corresponds to cells infected with JUNV during the first 16 h and then with JUNV-GFP during the last 8 h. Axis corresponds to the side scatter parameter (SSC) and the fluorescence intensity of JUNV GPC-A647. The highlighted regions indicate the gating used to distinguish non-infected (Non inf) from infected cells (total) and cells that expressed relatively high amounts of GPC (high). (**c**) Among the populations gated in (**b**), cells were further analysed for their GFP content. The axis corresponds to the side scatter parameter (SSC) and the GFP fluorescence intensity corresponding to the second round of infection. (**d,e**) Histogram representation comparing the fraction of cells expressing JUNV-GFP in cells that had not been infected at all (−1^st^ infection, −2^nd^ infection), cells infected only with JUNV (+1^st^ infection) using a MOI of 0.5 (**d**) or 2 (**e**), cells incubated only with JUNV-GFP at MOIs ranging from 0.03 to 0.1 during the second round of infection (−1^st^ infection, +2^nd^ infection) and cells incubated first with JUNV and then with JUNV-GFP (+1^st^ infection, +2^nd^ infection). Data correspond to mean ±SD from two independent experiments equivalent to the one shown in (**b**,**c**) performed in duplicate. At least 10,000 cells were acquired per sample. (**f**) JUNV-GFP–positive cells (GFP+ cells) from cells that were infected (GPC-A647 positive) or not (GPC-A647 negative) during the first round were further analysed for their GFP mean fluorescence intensity levels. The histogram shows similar GFP fluorescence intensity in both populations.

Because the Z protein of the OW arenavirus LCMV has also been suggested to inhibit translation^22^, we took advantage of the JUNV/JUNV-GFP superinfection system (Fig. 4a) to investigate effects during superinfection on viral translation at the single cell level (Fig. 4). Flow cytometry allowed discrimination of Vero cells infected during the first round (GPC antibody staining; Fig. 4b) and the second round (GFP protein expression; Fig. 4c). Prior JUNV infection had no effect on the number of GFP-expressing cells, even when taking into account only the highest GPC-expressing cells from the first infection (Fig. 4d) or using higher MOI (Fig. 4e). Moreover, cells infected during the second round (GFP+ cells) exhibited similar GFP fluorescence intensity regardless of whether they were infected (GPC-A647 positive) or not (GPC-A647 negative) during the first round of infection (Fig. 4f). These results suggest that JUNV is fully capable of superinfection.

Overall, our study shows that acutely infected cells remain permissive to a second round of JUNV infection to the same extent as non-infected cells. Our JUNV/JUNV-GFP detection system allowed us to monitor acute infection and readily discriminate between the first and second rounds of infection. We note that it was not feasible to determine with this assay whether the newly formed JUNV-GFP particles were infectious because they lacked GPC, which was replaced by GFP; thus, they were unable to bind to the natural receptor in the host cell.

Previous studies have shown that JUNV can induce superinfection exclusion, but these experiments by Ellenberg *et al.* were performed in cells chronically infected by JUNV for several years^10^,^11^. In one of these reports^10^, the authors suggested that failure to superinfect chronically infected Vero cells was related to the presence of NP and proposed that inhibition could occur at a step between replication and translation^10^. In the second study^11^, Ellenberg *et al.* explained superinfection exclusion by the fact that chronically infected BHK-21 cells diminished synthesis of superinfecting virus proteins, along with an inhibition of JUNV budding mediated by the overexpression of Tsg101^11^. In contrast, our superinfection experiments were exclusively conducted during acute infection of Vero or A549 cells. We showed that viral entry and viral genome replication and protein synthesis were normal or even slightly higher during the second round of infection. One possible explanation for the differences between our findings with acutely infected cells and those that Ellenberg *et al.* obtained for chronically infected Vero cells^12^ is that the cells they used do not express GPC or produce infectious particles and are resistant to the cytopathic effects of Junin virus, suggesting that failure to superinfect might be related to a host perturbation. Nevertheless, their results in BHK-21 chronically infected cells^13^ are consistent with our own observation with acutely infected Vero cells, showing that viral transcription and translation were not perturbed during superinfection. Although particle assembly and release was diminished in the chronically infected BHK-21 cells, it remains to be determined whether a similar perturbation is manifested in the acutely infected Vero cells.

In conclusion, our results highlight an important mechanistic difference in superinfection exclusion, not only between the Junin virus and other closely related OW arenaviruses^11^ but also with the large number of other virus family members that show superinfection exclusion.

## Methods

### Reagents

The following antibodies targeting JUNV proteins were first characterized in^14^ and were provided by BEI Resources: SA02 mouse monoclonal anti-NP antibody (2 μg ml^−1^; SA02-BG12; NR-2573), GB03 mouse monoclonal anti-GPC antibody (2 μg ml^−1^; QB06-BE08; NR-2564), LD05 mouse monoclonal anti-GPC antibody (2 μg ml^−1^; LD05-BF09; NR-48833), and GD01 mouse monoclonal anti-GPC antibody (2 μg ml^−1^; GD01-AG02; NR-43776). Antibodies were coupled to Alexa Fluor 488, 568, or 647 carboxylic acid (succinimidyl ester).

### Cell maintenance

All cells were maintained at 37 °C and 5% CO_2_. Vero cells (ATCC) and BSR-T7 were cultured in Dulbecco’s Modified Eagle’s medium (DMEM), GlutaMAX (Gibco), and A549 cells (ATCC) were grown in DMEM/F-12, GlutaMAX (Gibco). Both media were supplemented with 10% heat-inactivated foetal bovine serum (Atlanta Biologicals), 100 IU ml^−1^ penicillin, and 100 mg ml^−1^ streptomycin (Gibco). Virus media corresponded to DMEM GlutaMAX supplemented with 2% heat-inactivated foetal bovine serum (Atlanta Biologicals), 100 IU ml^−1^ penicillin, and 100 mg ml^−1^ streptomycin (Gibco).

### Virus production and labelling

Junin virus or JUNV refers to the non-pathogenic vaccine strain *Candid1* (obtained from the Bavari laboratory at the US Army Medical Research Institute of Infectious Diseases). Wild-type JUNV was produced and labelled as previously described^15^. Briefly, JUNV stock was incubated with the non-neutralizing mouse monoclonal antibody LD05 raised against the JUNV envelope glycoprotein (4 μg ml^−1^) and coupled to Alexa Fluor 647 (Life Technologies) for 30 min at 25 °C. The virus–dye mixture was then gently applied on top of a 10% Optiprep (Sigma-Aldrich) cushion and ultracentrifuged at 150,000 ×*g* for 2 h. The pellet containing the JUNV-A647–labelled particles was resuspended in virus media. The JUNV-GFP virus was produced as follows: BSR-T7 cells were infected with the vTF7-3 vaccinia virus^21^ for 1 h and then simultaneously transfected with the plasmids pJCd1L, pJCd1S-DGPC:GFP, and pC-GPC as described in^20^, using TransIT-2020 Transfection Reagent (Mirus). After 5 h, cells were washed and re-incubated in virus media.

### Immunofluorescence and light microscopy

Vero cells grown on coverslips, infected or non-infected, were fixed with 4% paraformaldehyde and incubated (without permeabilization) with the GB03-A568 antibody specifically recognizing the glycoprotein GPC of JUNV. Then, cells were fixed, permeabilized with 0.5% bovine serum albumin and 0.05% saponin in phosphate-buffered saline, and stained with the mouse monoclonal anti-NP antibody SA02-A647 and 4’,6-diamidino-2-phenylindole (DAPI). The samples were mounted on glass slides and imaged on a Zeiss AxioObserver.Z1 inverted microscope mounted with a spinning disc head (Yokogawa), a QuantEM:512SC EMCCD camera (Photometrics), and a 63 × 1.4 NA oil objective (Zeiss). Each acquisition corresponded to stacks spaced by 0.5 μm that spanned the whole cell volume, and images were analysed using ImageJ (version 1.48d).

### RNA analysis

Total RNA from cells was purified using the RNeasy Mini kit (Qiagen). RNA was then reverse-transcribed into complementary DNA using the SuperScript VILO cDNA Synthesis Kit (Life Technologies). RT-qPCR amplification was performed using FastStart Universal SYBR Green Master (Rox) (Roche). Complementary DNA of viral GPC, GFP, and glyceraldehyde 3-phosphate dehydrogenase (GAPDH) was detected using the following specific forward and reverse primers (5′→3′), respectively: viral GPC, cctagcgcttgcaggaagatcc and caccagctcatatctgctggatg; GFP, aagctgaccctgaagttcatctgc and cttgtagttgccgtcgtccttgaa; human transferrin receptor protein 1 (TFRC) caagctagatcagcattctctaacttg and cacatgactgttatcgccatctact; calcium voltage pump component (CACNA2D1) aagaccttgtcacactggca and agctggcgtgcattatttgg; and GAPDH, gagtcaacggatttggtcgt and ttgattttggagggatctcg.

PCR was performed on a StepOnePlus Real-Time PCR System (Applied Biosystems), and amplification cycles were set as follows: PCR initial activation step, 10 min at 95 °C; 40 cycles of denaturation and combined annealing/extension, 15 s at 95 °C; and 1 min at 60 °C. Fluorescence data collection was achieved at the end of each cycle, and a melting curve showing primer specificity was included at the end of each reaction.

### Transferrin uptake

Transferrin uptake assay was performed as in^23^. Briefly, Vero cells were pre-incubated at 4 °C or 37 °C for 15 min followed by incubation for 10 min and at the same temperatures with 5 μg ml^−1^ Transferrin-Alexa Fluor 647 (Tf-A647, Life Technologies). After incubation, the 37 °C samples were cooled on ice and rinsed with cold PBS, and 4 °C and 37 °C samples were briefly incubated twice with 150 mM NaCl, 1 mM MgCl_2_, 0.125 mM CaCl_2_, 0.1 M glycine pH 2.5 to remove the surface bound Tf-A647. A sample incubated at 4 °C with Tf-A647 but not treated with the acid wash was used to estimate the amount of fluorescent transferrin bound at the cell surface. Cells were then resuspended in 200 μl of PBS containing 1% bovine serum albumin and 0.5 mM EDTA at 4 °C. Measurement of the fluorescence intensity, reflecting the extent of Tf endocytosis for each cell, was determined by flow cytometry using the 633 nm laser line of the FASCSCanto II (BD Biosciences).

### Virus infectivity assays

JUNV-infected cells were identified by flow cytometry. For flow cytometry, cells were trypsinized, fixed with 4% paraformaldehyde, and permeabilized with 0.5% bovine serum albumin and 0.05% saponin in phosphate-buffered saline, followed by incubation with a GD01 mouse monoclonal antibody (2 μg ml^−1^) specific for the JUNV glycoprotein and coupled to Alexa Fluor 647. Acquisition of fluorescence intensity of the infected cells was performed on a FACSCanto II (BD Biosciences) using 488 nm (for GFP detection) and 640 nm (for Alexa Fluor 647 detection) lasers. Analysis of the percentage of infected cells was completed using FlowJo (Treestar Inc).

## Additional Information

**How to cite this article**: Gaudin, R. and Kirchhausen, T. Superinfection exclusion is absent during acute Junin virus infection of Vero and A549 cells. *Sci. Rep.* **5**, 15990; doi: 10.1038/srep15990 (2015).
